# Umbelliferone as an effective component of Rhodiola for protecting the cerebral microvascular endothelial barrier in cSVD

**DOI:** 10.3389/fphar.2025.1552579

**Published:** 2025-03-17

**Authors:** Li Tang, Hongfa Cheng, Qiuyue Yang, Yahui Xie, Qiuxia Zhang

**Affiliations:** College of Traditional Chinese Medicine, Capital Medical University, Beijing, China

**Keywords:** Rhodiola, umbelliferone, cSVD, network pharmacology, molecular docking, traditional Chinese medicine

## Abstract

**Objective:**

Rhodiola is a common Chinese herb in the treatment of cerebral small vessel disease (cSVD). Umbelliferone, one of the effective components of Rhodiola, can protect the endothelial barrier. But its mechanisms are still unclear. Therefore, this study is aimed to explore mechanisms of umbelliferone of an effective component of Rhodiola in protecting the cerebral microvascular endothelial barrier in cSVD.

**Methods:**

Firstly, ETCM, SwissTargetPrediction and literatures were used to screen components and targets of Rhodiola. GeneCards was used to obtain targets of cSVD. STRING and Cytoscape were utilized for building the PPI and C-T network. Metascape was utilized to construct GO and KEGG enrichment analysis. Then, molecular docking was employed to evaluate the binding ability of the compounds for their respective target molecules. Ultimately, the endothelial cell damage caused by OGD was employed to explore the protective impact of umbelliferone, a bioactive constituent of Rhodiola, on the endothelial barrier. Endothelial cell leakage and migration assays were used to assess the permeability and migration ability of endothelial cells. IF and WB techniques were employed to ascertain the expression of endothelial tight junction protein. The major target proteins and related pathways were validated by WB.

**Results:**

Six effective components and 106 potential targets were identified and 1885 targets of cSVD were obtained. Nine key targets were selected. GO and KEGG enrichment analysis suggested that effects of Rhodiola in cSVD were associated with PI3K-Akt, Ras, Rap1 and MAPK signal pathways. Molecular docking results showed good binding ability between 28 pairs of key proteins and compounds. Umbelliferone of an effective component of Rhodiola can protect tight junction proteins and improve the permeability and migration ability of endothelial cells damaged by OGD through MMP9, MMP2, CCND1, PTGS2 and PI3K-Akt, Ras, Rap1 signaling pathways.

**Conclusion:**

Our study systematically clarified mechanisms of Rhodiola in treating cSVD by network pharmacology and molecular docking, characterized by its multi-component, multi-target and multi-pathway effects. This finding was validated through *in vitro* tests, which demonstrated that umbelliferone of an effective component in Rhodiola can protect the brain microvascular endothelial barrier. It provided valuable ideas and references for additional research.

## Highlights

• Systematicness of mechanisms of Umbelliferone, an effective component of Rhodiola, on the cerebral microvascular endothelial barrier
• The first systematic study on the network pharmacology of Rhodiola in treating cSVD
• Validated that Umbelliferone can protect endothelial cells through multiple targets and pathways

## 1 Introduction

Cerebral small vessel disease (cSVD) is used to describe a group of neuroimaging and neuropathological abnormalities that are found in the white matter and deep gray matter of the brain (Hamilton et al., 2021). Small penetrating cerebral vessels, including arterioles, venules, and capillaries are affected by its pathological processes (Bordes et al., 2022). Adults over 55 are more likely to have intracerebral hemorrhage (ICH) caused by this disorder (Gurol et al., 2020). The progression of cSVD is shaped by a complicated blend among heritable and external influences (Greenberg, 2006), making it extremely challenging to manage. Therefore, it is imperative to find effective medicines to treat cSVD.

Rhodiola, also known as Hong Jingtian, is a member of the genus Rhodiola in the family Crassulaceae (Wang L. et al., 2021), which is a common traditional Chinese herb with qi invigorating and blood circulation promoting effects. Rhodiola was recorded in *Shennong’s Classic of Materia Medica* (Wang et al., 2019). 96 species of Rhodiola have been discovered globally, with the majority concentrated in diverse geographic areas within China (Wang L. et al., 2021). The medicinal variety of Rhodiola listed in the *Chinese Pharmacopoeia* is Rhodiola crenulate (Wang et al., 2019). Rhodiola has multiple functions encompassing anti-inflammatory, antioxidant, anti-viral, immunoregulatory, anti-fatigue, and improvement of learning and memory, with umbelliferone emerging as a pivotal effective component (Barbagallo et al., 2018; Li et al., 2022; Wang L. et al., 2021). Umbelliferone, a natural coumarin derivative, has gained significant attention for its potent pharmacological activities, including antioxidant and anti-inflammatory effects (Wu et al., 2022). Additionally, it possesses favorable ability to cross the blood-brain barrier (BBB), making it a promising candidate for targeting cerebrovascular disorders (Wang et al., 2015). In particular, umbelliferone has been shown to protect endothelial cells from damage induced by oxidative stress, making it a promising candidate for addressing endothelial dysfunction in cSVD (Zhang et al., 2023). Therefore, this study aims to explore the mechanisms of umbelliferone, a pivotal effective component of Rhodiola, in protecting the cerebral microvascular endothelial barrier, which is a critical aspect of cSVD pathogenesis.

Network pharmacology represents a new science that employs principles of systems biology and bioinformatics (Goh et al., 2007; Liu J. et al., 2021). Its characteristics of systematicity and integrity are consistent with the holistic view inherent to traditional Chinese medicine (TCM) (Li and Zhang, 2013; Lin et al., 2021). Therefore, the technology of network pharmacology is currently being increasingly applied in TCM research. In TCM, the network pharmacology reveals the “drug-protein-disease” interaction (Mangione et al., 2020), thus systematically explaining the complex relationship between TCM and disease (Hopkins, 2008). Moreover, molecular docking represents an instrumental methodology utilized to elucidate the recognition and interaction of small molecule ligands with protein receptors by predicting the affinity intensity and binding mode (Alonso et al., 2006; Koukos et al., 2021). In contemporary times, there has been a growing application of the integration of molecular docking technology and network pharmacology in the field of TCM. This has provided innovative insights into the mechanisms of action of TCM herbal remedies (Fotis et al., 2018; Zhang et al., 2019).

**FIGURE 1:**
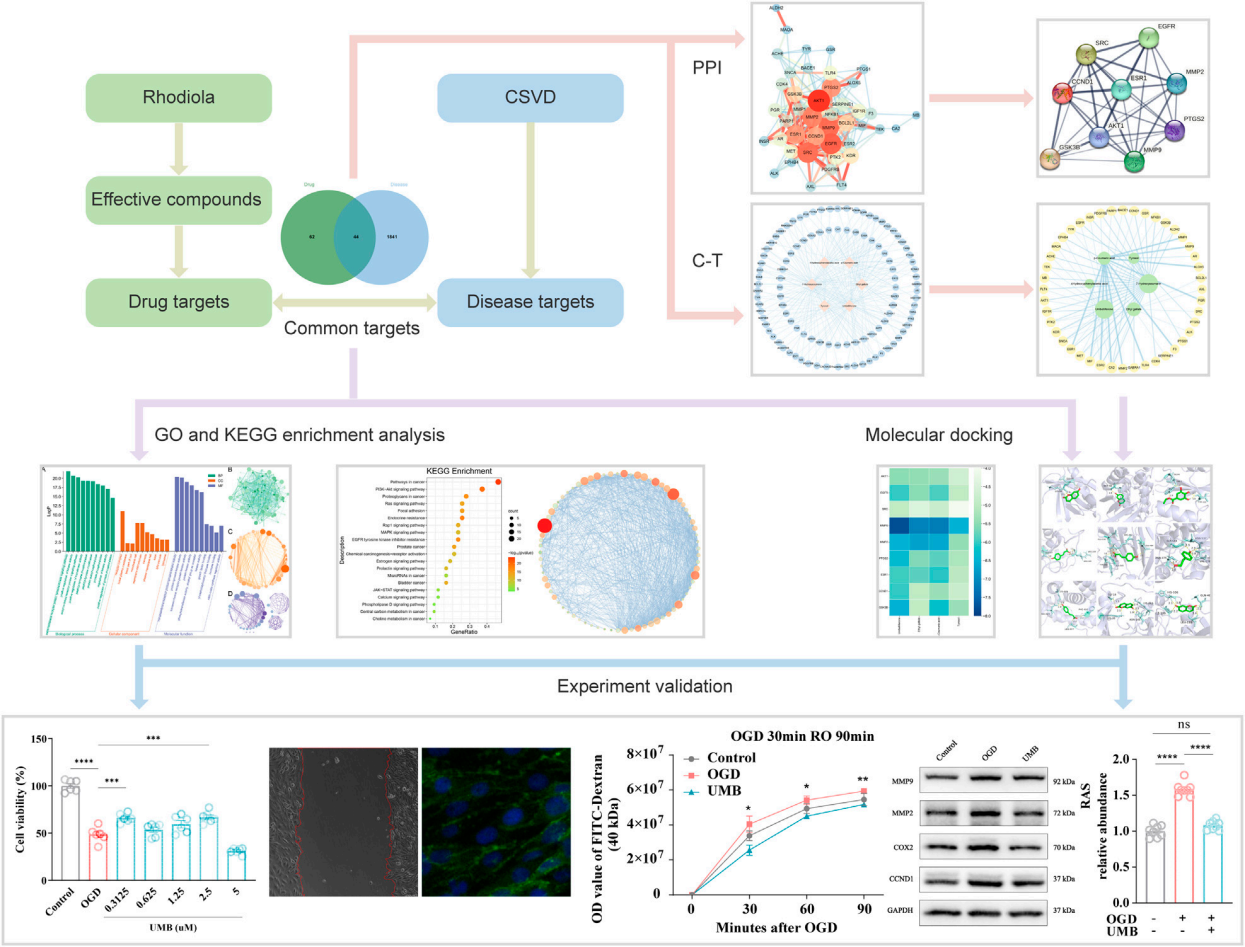
Graphical abstract of the study procedure using network pharmacology, molecular docking and experimental verification.

As far as we know, the study represents the inaugural investigation into the pharmacological mechanisms of Rhodiola in treating cSVD employing molecular docking technology and network pharmacology. The biological process was subjected to analysis using network pharmacology, with the results subsequently verified through cell experiments and molecular docking. Our study explored the effective components, potential targets and molecular mechanisms of Rhodiola against cSVD. These findings contribute to the related field of basic research and clinical treatment (Figure 1).

## 2 Methods and materials

### 2.1 Component screening and target prediction of Rhodiola

Initially, the candidate components of Rhodiola were obtained by the, ETCM (http://www.tcmip.cn/ETCM/index.php/Home/Index/) database and literature search (Ma et al., 2022; Xu et al., 2019). Subsequently, the Swiss ADME system (http://www.swissadme.ch/) was employed to ascertain data regarding the absorption, distribution, metabolism and elimination of drug components (Daina et al., 2017). Finally, the effective compounds were selected based on the criteria GI absorption as “high” and BBB permanent as “yes” (He et al., 2022). Given the close relationship between the pathogenesis of cSVD and blood-brain barrier (BBB) leakage, it is essential that the effective components to be screened possess the capacity to pass through the BBB (Thrippleton et al., 2019).

The initial step involved searching for standardized SMILES of efficacious constituents within the PubChem database (https://pubchem.ncbi.nlm.nih.gov/) to obtain their standardised SMILES (Kim et al., 2021). Subsequently, the standardized SMILES of the effective components were submitted to the SwissTargetPrediction platform, with the species selected as “*Homo sapiens*” (Daina et al., 2019). Afterwards, the predicted targets of the efficacious constituents of Rhodiola were downloaded, screened and summarised. Moreover, the reliability of these compounds in predicting targets was validated through the literature search.

### 2.2 Protein target prediction of cSVD and common targets between Rhodiola and cSVD

GeneCards (https://www.genecards.org/) refers to a comprehensive and authoritative database of annotative information about human genes (Safran et al., 2010), which enabled the prediction of disease targets of cSVD. The disease target information for cSVD was collated and downloaded using the keyword “cerebral small vessel disease” as a search term. Subsequently, the Excel software was employed to remove the redundant targets on three occasions, with the filter condition set to ≥ the median of “Relevance score”. Accordingly, the disease targets of cSVD were identified. Afterwards the Jvenn (http://www.bioinformatics.com.cn/static/others/jvenn/example.html) website was utilized for the purpose of identifying shared targets of Rhodiola and cSVD (Bardou et al., 2014).

### 2.3 The construction of protein-protein interaction (PPI) and “component-target” (C-T) networks

In order to further study the interactions between common targets, the PPI network was constructed using the STRING (https://cn.string-db.org/) database, utilizing data from both experimental verification and prediction (Szklarczyk et al., 2019). Firstly, the commonly targeted genes underwent importation to the STRING database. Secondly, the screening conditions were set as follows: the species was selected as “*H. sapiens*”, while the remaining settings were left at their default values. A minimum interaction score of ≥0.400 was established as the requisite threshold for medium confidence (Gan et al., 2021). Subsequently, the results obtained via the STRING database underwent importation into Cytoscape 3.9.1 in order to facilitate visualisation. The topological attributes, such as Degree, Closeness Centrality (CC), and Betweenness Centrality (BC), were calculated via the “network analysis” function of Cytoscape to identify (Gu et al., 2013). Afterwards, the color, size, transparency and other parameters of “Node” and “Edge” were modified through the “style” of Cytoscape. Finally, the PPI visualization image was exported.

Cytoscape is a software program designed for network biology visualization and analysis, illustrating the interactions between molecules (Otasek et al., 2019; Shannon et al., 2003). The targets of effective components of Rhodiola were transferred to the Cytoscape 3.9.1 platform, where they were used in the construction of a “component-target” (C-T) network. Additionally, a topology analysis enabled the determination of the connection between the effective components and targets. This analysis enabled the identification of key components (Lin et al., 2021).

### 2.4 Enrichment analysis by gene ontology (GO) and kyoto encyclopedia of genes and genomes (KEGG)

Enrichment analysis of GO and KEGG was performed using the web-based portal Metascape (http://metascape.org) (Zhou et al., 2019). Maintained default significance parameters of P < 0.01, Min Enrichment ≥1.5, and Min Overlap ≥3 (Li et al., 2020). Four enrichment analyses were conducted respectively including GO biological processes (BP), GO cellular components (CC), GO molecular function (MF), and KEGG pathway. After that, the Bioinformatics (http://www.bioinformatics.com.cn/) website enabled the generation of visual representations of the dot bubble and GO term enrichment, based on the aforementioned results (Chen et al., 2021).

### 2.5 Molecular docking

The use of molecular docking can facilitate the prediction of the affinity and binding mode of protein-ligand complexes, thereby verifying the binding ability of pivotal constituents of Rhodiola and key proteins identified by network pharmacology (Chen et al., 2020). (1) Preliminary documentation of protein receptors and small molecule ligands: The key proteins’ three-dimensional structures can be found in the PubChem (https://pubchem.ncbi.nlm.nih.gov/) database, and their PDB format files were downloaded (Burley et al., 2021; Kim et al., 2021). In a similar manner, the small molecule structures underwent download in the TCMSP (https://old.tcmsp-e.com/tcmsp.php) database (Ru et al., 2014). (2) Pdbqt files of protein receptors and small molecule ligands: the obtained structures of proteins and small molecule components were respectively imported into PyMOL software to remove solvent and organic substances (Seeliger and de Groot, 2010). Then, the aforementioned results were imported into AutoDockTools1.5.7, where all hydrogens were added and the protein ligands and small molecule receptors were set, respectively (Morris et al., 2009). Subsequently, the results were exported in the pdbqt format. (3) Docking box setting: the pdbqt files were imported into AutoDockTools1.5.7, and parameters were set so that proteins could be fully encompassed by the docking box (Wang Y. et al., 2021). (4) Molecular docking and visualisation: AutoDock4 was employed to perform molecular docking of receptors and ligands (Santos-Martins et al., 2021). The resulting molecular docking data for protein receptors and their corresponding ligands were visualised through PyMOL (Martinez et al., 2019).

### 2.6 Culture of bEnd.3 cells

The bEnd.3 cells (mouse brain-derived endothelial cell line, the initial passage was P7) were kindly donated by the laboratory of Capital Medical University (ATCC, CRL-2299, Unites States). The cells received cultivation in a complete medium (CM), which consisted of Dulbecco’s Modified Eagle Medium (DMEM, Gibco, C11995500BT, Unites States), fetal bovine serum (FBS, Gibco, 35-081-CV, Unites States; 10%), penicillin (100 U/mL, Gibco, 15140-122, Unites States) and streptomycin (100 μg/mL, Gibco, 15140-122, Unites States). Upon achieving confluency of 80%–90% of the cells, passaging was performed.

### 2.7 Oxygen-glucose deprivation (OGD) model and treatment

Oxygen-glucose deprivation (OGD) treatment was conducted by replacing the medium with glucose-free DMEM (Gibco, 11966-025, Unites States) and transferring the bEnd.3 cells into a three-gas incubator (Thermo Fisher Scientific, Unites States) with oxygen (O_2_, 1%), carbon dioxide (CO_2_, 5%), and nitrogen (N_2_, 94%) at a temperature of 37°C for 12 h. Thereafter, the cells were transferred to complete DMEM/high glucose medium and cultured for a further 24 h under normoxic conditions. To assess the impact of UMB on the brain microvascular endothelial barrier, varying concentrations of UMB were introduced to the medium at the onset of reoxygenation.

### 2.8 Cell viability assay

bEnd.3 cells were grown at a density of 1 × 10^4^ cells/well) in a 96-well plate. Following the completion of the specified treatments, 10 µL of CCK-8 solution (Vazyme, A311-01, Nanjing, China) was pipetted into the wells. After 2 hours of incubation at 37°C, the optical density (OD) readings were taken utilizing a microplate reader.

### 2.9 Scratch test

bEnd.3 cells were added to 24-well plates with densities of 5 × 10^4^ cells/well. Subsequently, the control and OGD groups were cultured in fresh complete medium and DMEM medium without serum, respectively. The UMB group received 0.3 μM UMB. Following a 24-hour period of UMB treatment, a line was drawn across the cell monolayers with an equivalent force using a 10 μL pipette tip. Cells were subsequently gently washed using PBS. At 0, 12 and 24 h, images were captured using a microscope. Statistical analysis on cellular migration process was conducted by means of ImageJ program.

### 2.10 Western blot (WB)

The bEnd.3 cells were rinsed three times using PBS, and proteins were isolated by applying RIPA Lysis Buffer (NCM Biotech, WB3100, CN). Bicinchoninic acid (BCA) protein assays (Applygen, P1511, Beijing, China) were used to determine protein concentrations. The protein concentrations were determined using a bicinchoninic acid (BCA) protein assay kit. The primary antibodies used are listed below: ZO1 (Proteintech, 21773, Unites States, 1:5000), Occludin (Abcam, ab216327, United Kingdom, 1:1000), Claudin5 (Invitrogen, 35-2500, Unites States, 1:1000), Ras (Abcam, ab52939, United Kingdom, 1:5000), ERK (Cell Signaling Technology, 4695S, Unites States, 1:1000), p-ERK (Cell Signaling Technology, 4370S, Unites States, 1:2000), Raf (Santa Cruz Biotechnology, sc-7267, Unites States, 1:200), Mek (Santa Cruz Biotechnology, sc-17820, Unites States, 1:100), PI3K (Abcam, ab180967, United Kingdom, 1:2000), AKT1 (Santa Cruz Biotechnology, sc-5298, Unites States, 1:1000), p-AKT1 (Cell Signaling Technology, 4060T, Unites States, 1:2000), Rap1 (Santa Cruz Biotechnology, sc-53434, Unites States, 1:200), MMP9 (Proteintech, 10375, Unites States, 1:500), MMP2 (Santa Cruz Biotechnology, sc-13595, Unites States, 1:200), PTGS2 (Santa Cruz Biotechnology, sc-376861, Unites States, 1:100), CCND1 (Santa Cruz Biotechnology, sc-8396, Unites States, 1:200), and GAPDH (GeneTex, GTX100118, Unites States, 1:5000).

### 2.11 Immunofluorescence staining (IF)

The cultured cells were rinsed using PBS and then treated for fixation within 4% paraformaldehyde (PFA) at 15 min. After washing in PBS, cells were permeabilized in 0.5% TritonX-100 (Merck Millipore, T8787, St. Louis, MO, Unites States) within 20 min. Thereafter, cells were blocked by 5% bovine serum albumin (BSA, ServiceBio, GC305010, CN) during 30 min and incubated in the primary antibody (ZO1, Proteintech, 21,773, Unites States, 1:1000) at 4°C overnight. Next, the associated secondary antibodies were incubated at ambient temperature for an hour, then stained with DAPI (MedChemExpress, HY-D0814, Unites States) for 5 minutes. Images underwent visualization by means of a fluorescence camera microscope (Leica, Wetzlar, Germany), and positive expression quantification was performed by ImageJ program.

### 2.12 Leakage assay

The bEnd.3 cells were plated at a density of 5 × 10^4^ cells per well in a 24-well Transwell with PC membrane insert (Corning, 14311, Unites States). Following the attainment of 100% confluency, the cells were cultured for a period of 48 h to facilitate the formation of tight junctions. Subsequently, the cells were OGD-treated as previously described for a period of 12 h. Following this, FITC-dextran (25 mg/mL, 40 kDa, Chondrex, 4,009, Unites States) was added to the superior chamber. Ultimately, we measured the diffusion rate of the tracer from the top to the bottom chamber at 30, 60, and 90 min after the onset of OGD (Li et al., 2023).

### 2.13 Statistical analysis

The data underwent analysis and visualization via GraphPad Prism 9 software (GraphPad Software, San Diego, United States). The results were expressed as mean ± standard error of the mean (SEM). The Shapiro-Wilk test provided a means of determining whether the data exhibited a normal distribution. In the case of data that were normally distributed, a one-way analysis of variance (one-way ANOVA) was employed, with Tukey’s tests subsequently utilized to examine discrepancies pertaining to each of the designated groups. The p-value was deemed statistically significant when it was below 0.05.

## 3 Results

### 3.1 Umbelliferone (UMB) is the main effective ingredient of Rhodiola in treating CSVD

A total of 46 candidate constituents of Rhodiola were obtained via ETCM and through a process of literature verification. Subsequently, the candidate components were input into the SwissADME system and evaluated according to the following criteria: “high” gastrointestinal (GI) absorption and “yes” for blood-brain barrier (BBB) permanent. The screening process has identified six eligible effective components. The remaining components were identified as Tyrosol, Umbelliferone, 4-Hydroxyphenylacid acid, Ethyl gallate, 7-Hydroxycoumarin and p-Coumaric acid. The standardized SMILES of the six efficacious constituents were retrieved via the PubChem and transferred to the SwissTargetPrediction platform, with the selected species being “*H. sapiens*”. The six most effective compounds yielded a total of 226 predicted targets. Finally, any duplicates were removed by Microsoft Excel software, leaving 106 predicted targets of the effective compounds of Rhodiola (Supplementary Table S1).

**FIGURE 2:**
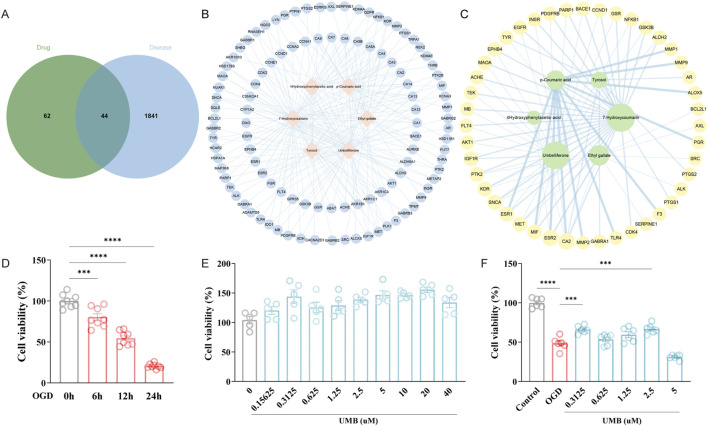
Umbelliferone (UMB) represents the primary effective ingredient of Rhodiola in treating CSVD. **(A)** Venn graph of 44 common targets of Rhodiola and cSVD. **(B)** The Rhodiola “component-target” (C-T) network. The nodes situated in the central region represent the effective compounds of Rhodiola, while the nodes positioned in the surrounding area represent the drug targets. **(C)** A network of connections between the effective compounds of Rhodiola and common targets. The size of a node is indicative of the critical importance of the compound represented by that node. **(D)** The CCK-8 assay was applied to determine cell viability following OGD for varying periods of time (6, 12, 24 h). **(E)** UMB was tested for cell viability using the CCK-8 assay at varying concentrations (0.15625 μM, 0.3125 μM, 0.625 μM, 1.25 μM, 2.5 μM, 5 μM, 10 μM, 20 μM, 40 μM) over a 24-h period. **(F)** Cell viability was determined with CCK-8 after UMB treatment (0.3125 μM, 0.625 μM, 1.25 μM, 2.5 μM, 5 μM) and OGD for 24 h. Data are shown as mean ± SEM **(D, F)**. ***p < 0.001 and ****p < 0.0001 vs. 0 h **(D)**. ***p < 0.001 vs. OGD and ****p < 0.0001 vs. control **(F)**.

A search and download of 15,087 disease targets of cSVD from the GeneCards database was conducted using the keyword “cerebral small vessel disease”. In the initial screening, 7,543 disease targets were identified with the median of “Relevance score” ≥7.336393. A second screening yielded 3,771 disease targets with the median of “Relevance score” ≥13.92903. Ultimately, 1,885 disease targets were selected based on a median “Relevance score” of ≥20.91511 (Supplementary Table S2). The 106 targets of effective compounds of Rhodiola and the 1885 targets of cSVD were input to the Jvenn website, respectively. This allowed the shared targets of the constituents and disease to be acquired and downloaded (Supplementary Table S3). As illustrated in Figures 2A, 44 Rhodiola anti-cSVD targets were obtained.

To examine the pharmacological action of Rhodiola, the efficacious constituents of Rhodiola and their respective targets were exported to Cytoscape 3.9.1, which was used to generate a C-T network (Figure 2B). The network comprises 112 nodes and 226 edges. The pink prismatic nodes situated in the central region of the network represent the six effective compounds of Rhodiola. The blue round nodes that surround them symbolize the component targets, and the blue edges illustrate the correlation between the effective constituents and targets. The key effective compounds were identified according to their Degree value, BC value and CC value (Figure 2C). The initial four compounds were selected for molecular docking. Further analysis revealed that Umbelliferone and 7-Hydroxycoumarin possess the same molecular structure. Consequently, Umbelliferone, Ethyl gallate, p-Coumaric acid and Tyrosol were identified as potential candidate compounds. The C-T network demonstrated that the pharmacological effects of Rhodiola exhibited multi-component and multi-target interactions.

To establish OGD models, bEnd.3 cells were incubated under anaerobic conditions (94% N_2_, 5% CO_2_, 1% O_2_) and in low-glucose media for varying periods of time. The cell culture exposed to OGD conditions for a period of 6–24 h exhibited a notable decline in cell viability, when compared to the control group. It is noteworthy that 12 h of cell culture under OGD conditions led to a considerable decline in cell viability, reaching 54.49% (slightly above 50%) (Figure 2D). Consequently, 12 h was selected as the modelling time for OGD. To exclude the toxic effect of UMB, the cell count kit-8 (CCK-8) assay was utilized to ascertain cell viability. The findings revealed that various concentrations of UMB (0.15625 μM, 0.3125 μM, 0.625 μM, 1.25 μM, 2.5 μM, 5 μM, 10 μM, 20 μM, 40 μM) had no cytotoxic effects on bEnd.3 cells (Figure 2E). The decline in cell viability resulting from OGD was counteracted by the administration of different concentrations of UMB, indicating that UMB has a preservative action towards OGD-induced damage to bEnd.3 cells. Among the concentrations tested, the greatest increase in cell viability was observed following treatment with 0.3125 μM UMB (Figure 2F). Consequently, subsequent experiments were conducted using a concentration of 0.3 μM for a 24-hour period.

### 3.2 UMB can improve cell migration ability and protect the cerebral microvascular endothelial barrier

**FIGURE 3:**
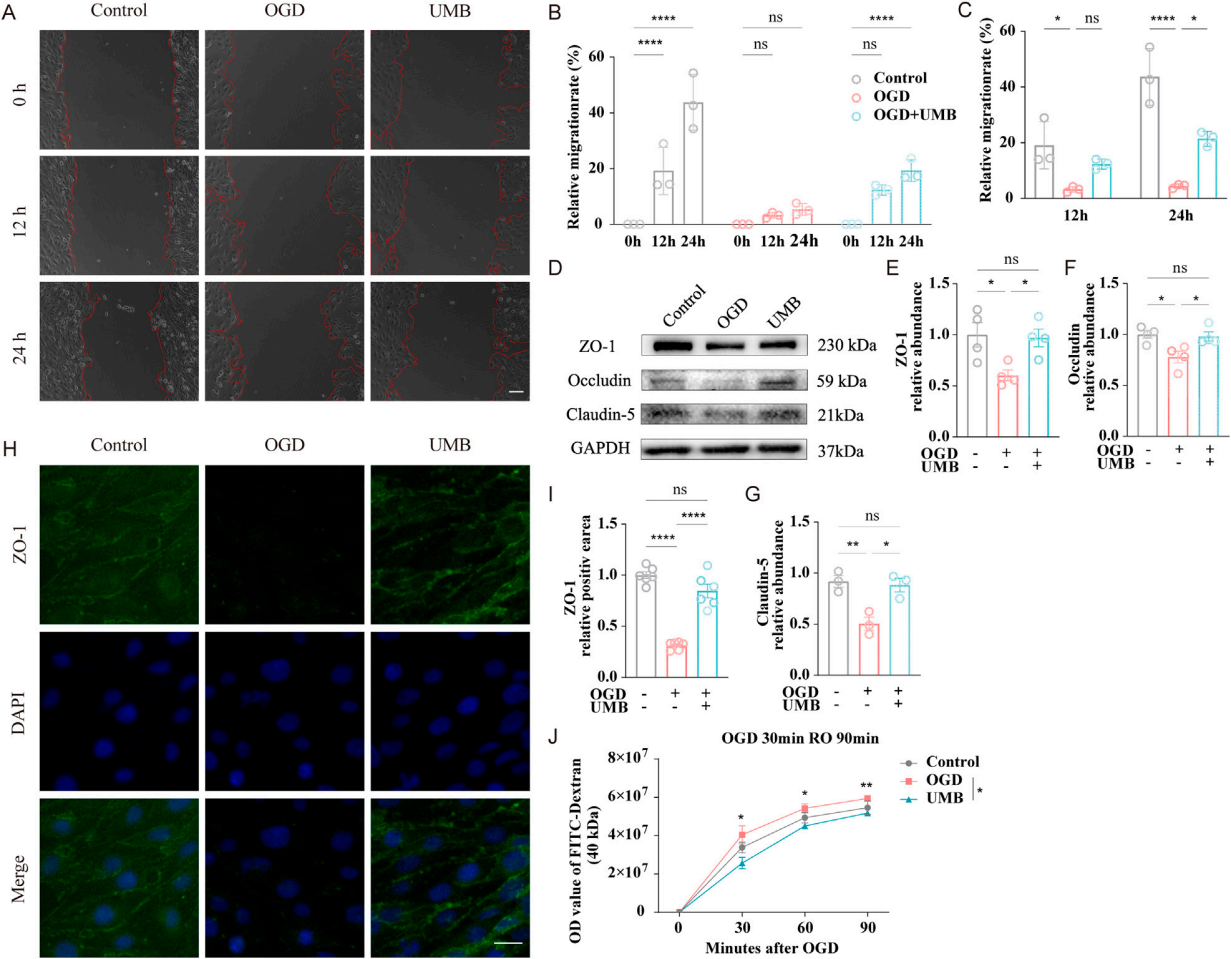
UMB can improve cell migration ability and protect the cerebral microvascular endothelial barrier. **(A)** Control, OGD, and UMB groups representative images at the beginning (0 h), middle (12 h), and end (24 h) of the wound healing assay. The red dashed lines delineate the region of cell migration. **(B)** Quantification of the relative migration rate in a wound healing assay of the control, OGD, and OGD + UMB groups after 0–24 h. **(C)** Quantification of the relative migration rate in a wound healing assay after 12 h and 24 h. **(D)** Western blot bands representative of ZO-1, occludin and claudin-5. **(E)** Relative abundance of ZO-1 protein expression. **(F)** Relative abundance of occludin protein expression. **(G)** Relative abundance of claudin-5 protein expression. **(H)** Representative micrographs of immunofluorescence staining for ZO-1/DAPI of bEnd.3 cells. The scale bar represents 50 μm (μm). **(I)** Representative quantitative analysis of ZO-1 within bEnd.3 cells. **(J)** The extent of BBB disruption was assessed by quantifying the extravasation of FITC-dextran dye into the bottom chamber at 0, 30, 60, and 90 min following OGD. Data are means ± SEM. ****p < 0.0001 vs. 0 h **(B)**. *p < 0.05, **p < 0.01, ***p < 0.001 and ****p < 0.0001 vs. control or OGD (others).

The capacity of bEnd.3 cells to migrate was evaluated through a wound healing assay. After 12 h of incubation, no evidence of cell migration was observed in the OGD and OGD + UMB groups. After 24 h, the relative migration rate for the OGD + UMB group was found to be markedly higher than that observed for the OGD group (Figures 3A–C). These findings indicate that UMB has the capacity to enhance the migratory capabilities of cells. To further substantiate the protective function of UMB on the cerebral microvascular endothelial barrier, Western blot, immunofluorescence staining, and leakage assay analyses were conducted. Western blot examination revealed a notable decline in the protein levels of ZO-1, occludin, and claudin-5 in the OGD group. However, incubation of bEnd.3 cells with UMB (0.3 μM for 24 h) yielded a notable reversal of these effects (Figures 3D–G). Furthermore, immunofluorescence staining demonstrated that UMB significantly enhanced the fluorescence intensity of ZO-1 when compared to the OGD group (Figures 3H, I). Moreover, the integrity of the BBB was assessed through a leakage assay. It was demonstrated OGD triggered dextran leakage starting from 30 min onwards, with the leakage further aggravated over time. A notable reduction in leakage was achieved by UMB (Figure 3J). The aforementioned results indicate UMB is capable of effectively safeguarding the cerebral microvascular endothelial barrier in bEnd.3 cells.

### 3.3 GO and KEGG enrichment analysis

**FIGURE 4:**
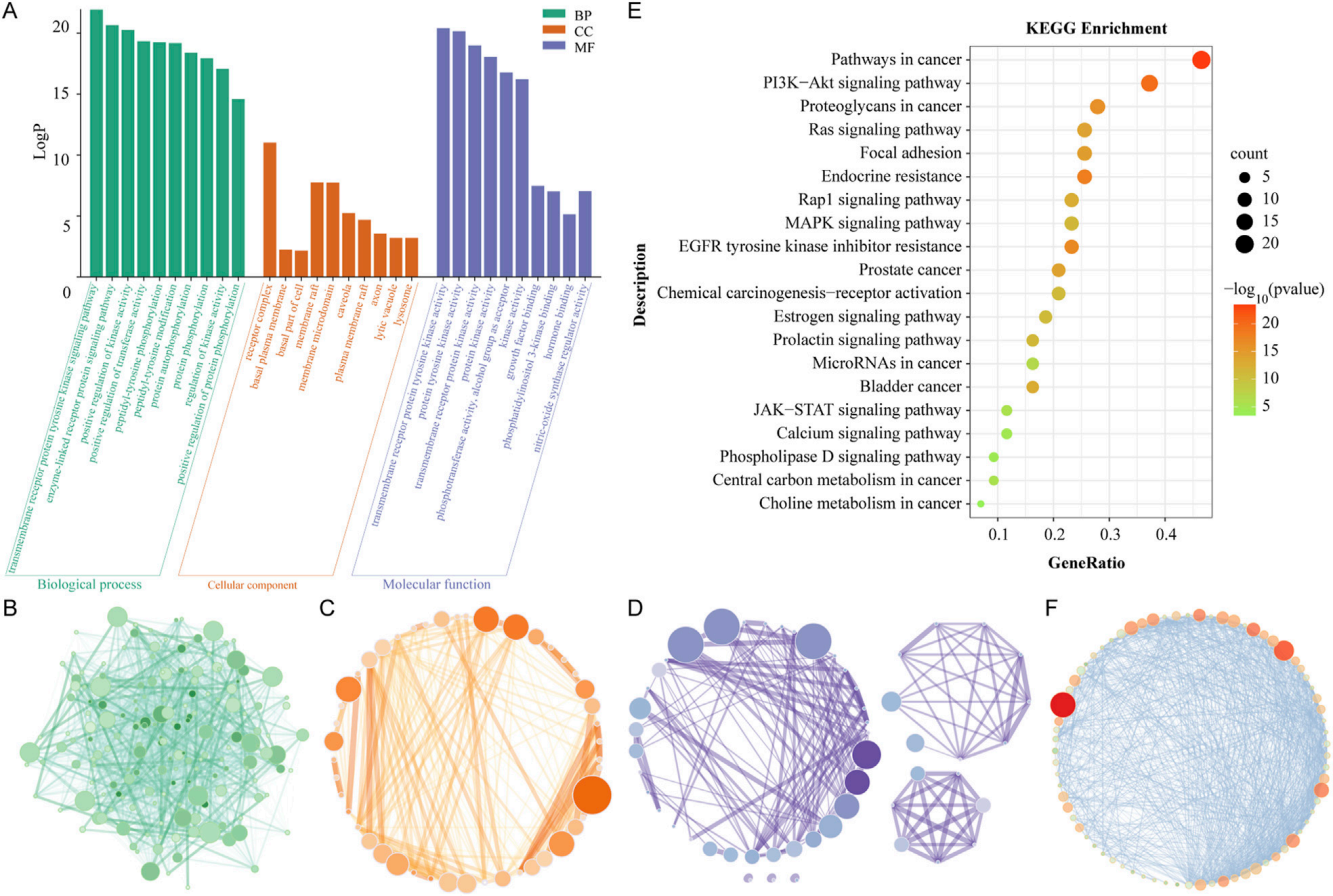
GO and KEGG enrichment analysis. **(A)** The diagram of enrichment GO terms, including those pertaining to BP, CC and MF. The X-axis represents the full names of the GO terms, while the Y-axis illustrates the LogP value. **(B)** GO network of BP. **(C)** GO network of CC. **(D)** GO network of MF. The magnitude and chromaticity of each node are indicative of the extent of enrichment for each GO term. **(E)** A diagram of the KEGG enrichment dot bubble. The X-axis depicts the gene ratios, while the Y-axis illustrates the descriptions of the primary signal pathways. The size and color of each bubble, respectively, indicate the gene count and the P value. **(F)** Visualization network of KEGG enrichment pathways. The magnitude and chromaticity of each node symbolize the extent of enrichment of the corresponding KEGG pathway.

GO enrichment analysis was performed to study the multifaceted biological effects of Rhodiola in treating cSVD. GO enrichment encompasses three categories: biological processes (BP), which encompass the functions realized by organisms through gene programming; cellular components (CC), which describe the relationships between gene functions and cell components; and molecular functions (MF), which pertain to the genes that regulate these functions. In this study, 744 GO terms were identified as enriched, including 635 BPs (Supplementary Table S4), 50 CCs (Supplementary Table S5) and 59 MFs (Supplementary Table S6). Based on the LogP scores, the top 10 significantly enriched terms were extracted from the three categories (Figure 4A) and visualized by network diagram (Figures 4B–D).

The results demonstrated the top 10 BPs as follows: transmembrane receiver protein tyrosine kinase signaling path, positive regulation of kinase activity, enzyme-linked receiver protein signaling path, positive regulation of transfer activity, pentyl tyrosine photosynthesis, pentyl tyrosine modification, protein autophysiology, protein photosynthesis, regulation of kinase activity, and positive regulation of protein phosphorylation. The results demonstrated that the biological programs of Rhodiola to treat cSVD were primarily mediated through transcriptional regulation, protein kinase activity, and protein phosphorylation. Top ten cellular components (CCs) were receiver complex, membrane raft, membrane microdomain, nuclear envelope, nuclear membrane, side of membrane, caveola, vascular men, and plasma membrane raft, as well as protein kinase complex. The results showed that Rhodiola may therapeutically affect cSVD through the receiver complex, the membrane raft, and the membrane microdomain. The top 10 MFs were as follows: transmembrane receiver protein tyrosine kinase activity, protein tyrosine kinase activity, transmembrane receiver protein kinase activity, protein kinase activity, photosensitive transfer activity, aluminum group as receptor, kinase activity, growth factor binding, nitric oxide synthase regulator activity, photosensitive linositol 3-kinase binding, kinase binding. It could be seen that the proteins of Rhodiola-cSVD were predominantly distributed in the categories of protein kinase activity and kinase binding.

A KEGG enrichment analysis was conducted to investigate the pathway mechanism of Rhodiola against cSVD. Supplementary Table S7 shows that 120 Rhodiola-cSVD-related pathways with statistical significance were identified through the Metascape web-based portal. The 20 pathways with the most significant enrichment were visualized by the enrichment dot bubble diagram (Figure 4E) and a network diagram (Figure 4F). The results showed that main signal pathways involved Pathways in cancer, PI3K Akt signaling pathway, Proteoglycans in cancer, Focal induction, Ras signaling pathway, Endocrine resistance, EGFR tyrosine kinase inhibitor resistance, Rap1 signaling pathway, MAPK signaling pathway, etc. It could be seen that the pharmacological effect of Rhodiola-cSVD was intimately related to the PI3K-Akt pathway.

### 3.4 UMB protects the cerebral microvascular endothelial barrier via the PI3K/AKT, RAP1 and RAS pathways

**FIGURE 5:**
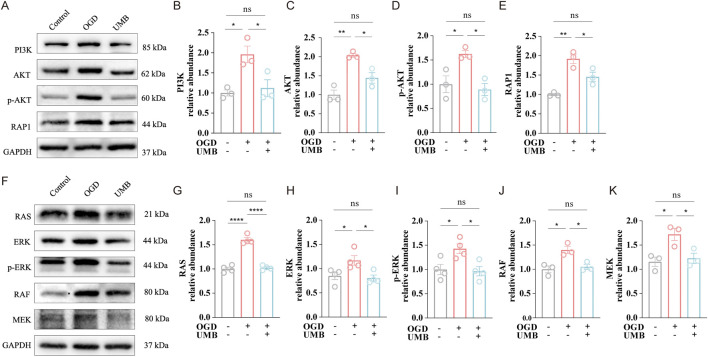
UMB protects the cerebral microvascular endothelial barrier via the PI3K/AKT, RAP1, and RAS pathways. **(A)** WB bands representative of PI3K, AKT, p-AKT and Rap1. **(B)** Relative abundance of PI3K protein expression. **(C)** Relative abundance of AKT protein expression. **(D)** Relative abundance of the p-AKT protein expression. **(E)** Relative abundance of Rap1 protein expression. **(F)** Representative Western blot bands of RAS, ERK, p-ERK, RAF and MEK. **(G)** Relative abundance of RAS protein level. **(H)** Relative abundance of ERK protein level. **(I)** Relative abundance of p-ERK protein level. **(J)** Relative abundance of RAF protein level. **(K)** Relative abundance of MEK protein level. Data are means ± SEM. *p < 0.05, **p < 0.01 and ****p < 0.0001 vs. control or OGD.

To substantiate the regulatory role of UMB on the aforementioned pathways, we conducted Western blot analysis. Data demonstrated the protein abundance of PI3K, AKT, p-AKT, and Rap1 was markedly elevated under OGD conditions. Conversely, UMB (0.3 μM for 24 h) potently suppressed the overexpression of major proteins in the PI3K/AKT and Rap1 pathways following OGD (Figures 5A–E). Similarly, Western blot testing indicated that UMB dramatically decreased the high expression of key proteins in the RAS pathway after OGD (Figures 5F–K). In conclusion, the data demonstrate that UMB protects the cerebral microvascular endothelial barrier through the PI3K/AKT, RAP1 and RAS pathways.

### 3.5 Protein-protein interaction (PPI) network analysis and molecular docking visualization

**FIGURE 6:**
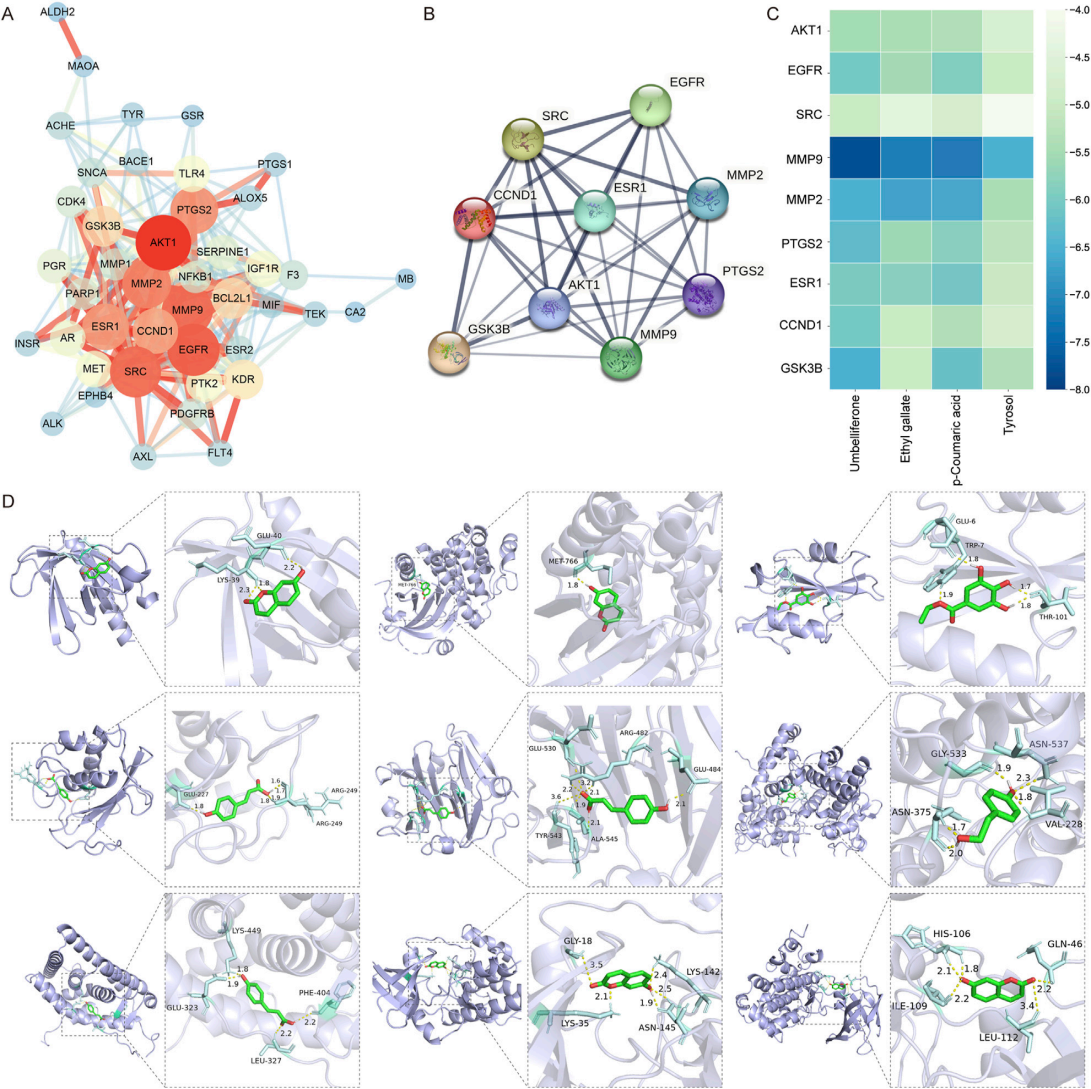
PPI network analysis and molecular docking. **(A)** PPI network of shared targets. The magnitude and chromaticity of a node indicate the likelihood of the protein being a key target. Similarly, the thickness and color intensity of an edge indicate the extent of the affinity between the two proteins connected by that edge. **(B)** PPI network analysis of nine key targets. **(C)** Heatmap of the molecular docking score. Thirty-six results of molecular docking between nine key targets (on the Y-axis) and four compounds (on the X-axis) were visualized in the heat map. The color blue indicates a lower binding energy, which corresponds to a higher combining ability between the ligand and receptor. **(D)** Diagram of molecular docking. The purple section is the protein receptor (target), while the green section is the ligand (compound). The enlarged diagram illustrates the names of amino acid residues and hydrogen bond length. The light blue section represents an amino acid residue, while the yellow line shows where a hydrogen bond is formed between the residue and a small molecular ligand.

The PPI network with 44 Rhodiola anti-cSVD proteins obtained via the STRING was constructed to further examine the interaction of common targets. These data were transferred to Cytoscape 3.9.1 for visualization purposes. After removing a disconnected node, a PPI network comprising 43 nodes and 280 edges was generated (Figure 6A). The top nine gene nodes were identified according to the Degree value: AKT1, EGFR, SRC, MMP9, MMP2, PTGS2, ESR1, CCND1 and GSK3B (Figure 6B). The degree values of the above nine nodes are 33, 30, 29, 29, 27, 26, 25, 24 and 20 respectively (Table 1). The results indicated that the interaction of these key targets may be utilized to elucidate the important pharmacological effects of Rhodiola against cSVD.

**TABLE 1:** Topology analysis data of the nine key targets.

Name	Degree	BetweennessCentrality	ClosenessCentrality
AKT1	33	0.153620355	0.807692308
EGFR	30	0.092616654	0.763636364
SRC	29	0.066267603	0.736842105
MMP9	29	0.100585574	0.724137931
MMP2	27	0.04848748	0.7
PTGS2	26	0.071301672	0.711864407
ESR1	25	0.03399481	0.666666667
CCND1	24	0.022790213	0.65625
GSK3B	20	0.028477657	0.636363636

In order to ascertain the bonding ability of the identified Rhodiola key constituents and cSVD core proteins, molecular docking to predict the bonding energy of protein-ligand complexes was used. According to the findings of network pharmacology, nine key proteins (AKT1, EGFR, SRC, MMP9, MMP2, PTGS2, ESR1, CCND1, GSK3B) and four key compounds (Umbelliferone, Ethyl gallate, p-Coumaric acid, Tyrosol) were employed as molecular docking targets. Results were presented visually as a heat map (Figure 6C). The models of the molecular docking of the nine core proteins and their associated components, along with the names given to the amino acid residues and the length of the hydrogen bonds, are illustrated in Figure 6D. The respective interactions between the key proteins and their corresponding compounds are as follows: AKT1 (PDB ID: 1UNQ) - Umbelliferone, EGFR (1XKK) - Umbelliferone, SRC (1O43) - Ethyl gallate, MMP9 (4XCT) - p-Coumaric acid, MMP2 (1GEN) - p-Coumaric acid, PTGS2 (5F19) - Tyrosol, ESR1 (1XP1) - p-Coumaric acid, CCND1 (2W96) - Umbelliferone and GSK3B (1O9U) - Umbelliferone.

The binding energy of −4.25 kcal/mol, −5.0 kcal/mol, or −7.0 kcal/mol, suggests that the ligand-receptor bonding activity was certain, good, or strong, respectively (Li C. et al., 2021; Li X. et al., 2021; Zeng et al., 2021). Moreover, a lesser bonding energy suggests a steadier receptor-ligand bond. Twenty-eight pairs of ligands demonstrated favorable binding activity with receptors among the 36 docking results. Among them, the ligands and receptors with strong binding activity are MMP9 and Umbelliferone (−7.78 kcal/mol), MMP9 and p-Coumaric acid (−7.19 kcal/mol), MMP9 and Ethyl gallate (−7.14 kcal/mol). The findings suggest that MMP9, a primary target of cSVD, and three prominent compounds from Rhodiola (Umbelliferone, p-Coumaric acid, Ethyl gallate) appear to exert an important impact on the management of cSVD.

### 3.6 UMB protects cerebral microvascular endothelial barrier via key targets of MMP9, MMP2, PTGS2 and CCND1

**FIGURE 7:**
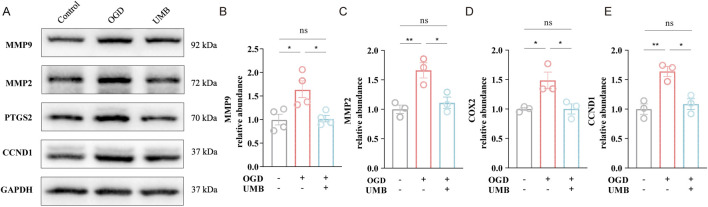
UMB protects cerebral microvascular endothelial barrier via key targets of MMP9, MMP2, PTGS2 and CCND1. **(A)** Representative Western blot bands of MMP9, MMP2, PTGS2 and CCND1. **(B)** Relative abundance of MMP9 protein expression. **(C)** Relative abundance of MMP2 protein expression. **(D)** Relative abundance of PTGS2 protein expression. **(E)** Relative abundance of CCND1 protein expression. Data are means ± SEM. *p < 0.05, **p < 0.01 and ***p < 0.001 vs. control or OGD.

To prove the effect of UMB on the key targets screened by PPI network analysis and molecular docking, as previously mentioned, Western blot assay was performed. These findings clearly suggested the protein levels of MMP9, MMP2, PTGS2 and CCND1 were elevated in the OGD conditions, whereas UMB (0.3 μM for 24 h) demonstrated a capacity to reduce the overexpression of key proteins, including MMP9, MMP2, PTGS2 and CCND1 (Figures 7A–E). On the basis of these findings, it can be concluded that UMB protects the cerebral microvascular endothelial barrier through key targets of MMP9, MMP2, PTGS2 and CCND1.

## 4 Discussion

CSVD is a prevalent neurological disorder among the elderly, exhibiting a gradual increase in prevalence (Lam et al., 2023; Shi and Wardlaw, 2016). It is an ordinary consequence of the aging process, representing a significant health risk for the elderly population (Wardlaw et al., 2013). CSVD is a complex, multifactorial disorder involving endothelial dysfunction, BBB breakdown, inflammation, and white matter lesions (Gao et al., 2022). Endothelial cell dysfunction, resulting from oxidative stress and impaired tight junction protein expression, plays a central role in the pathogenesis of cSVD (Lu et al., 2021). Rhodiola, a traditional Chinese herb, for decades has been employed clinically throughout China (Li and Su, 2018). However, the exact molecular mechanism through which Rhodiola produces its actions against cSVD remains unclear. Accordingly, our study focused on the potential role of Umbelliferone, an active component of Rhodiola, in protecting the cerebral microvascular endothelial barrier, which is a crucial step in the early stages of cSVD.

In this study, key components, including Tyrosol, Umbelliferone, Ethyl gallate, and p-Coumaric acid, were identified from 46 potential components of Rhodiola through, ETCM and Swiss ADME databases, as well as through a process of literature verification. For example, Tyrosol has the capacity to enter the brain and maintain a high concentration level, which enables the improvement of BBB permeability (Fan et al., 2020). Umbelliferone, a natural bioactive coumarin derivative, has been demonstrated to have antioxidant and anti-inflammatory effects (Kwak et al., 2019). For example, umbelliferone has been proven to inhibit rheumatoid arthritis invasion, migration, and inflammatory response via inhibiting the Wnt/β-catenin signal pathway (Cai et al., 2022). Ethyl gallate refers to a phenolic compound with proven anti-cancer and antioxidant effects (Liu et al., 2019). The evidence indicates that the therapeutic effect is exerted via the HIF-1α/EPO/VEGFA signaling pathway with regard to ischemic stroke (Zhang et al., 2022). In addition, p-Coumaric acid is a naturally occurring metabolite that has been demonstrated to mitigate inflammatory responses and oxidative stress (Boo, 2019). It can be reasonably inferred that these compounds of Rhodiola may have potential effects in the treatment of cSVD, which requires further investigation.

The most promising proteins from the 44 common targets of Rhodiola for the treatment of cSVD were identified through the implementation of a PPI network. Nine core proteins were selected based on their centrality within the network. The selected proteins are AKT1, EGFR, SRC, MMP9, MMP2, PTGS2, ESR1, CCND1, and GSK3. Some of these proteins have been previously associated with the pathogenesis of cSVD. For example, Akt has been demonstrated to protect endothelial cell barriers through oxidized phospholipids, thereby reducing vascular permeability (Singleton et al., 2009; Liu J. Y. et al., 2021) have proposed that rare mutations in NOTCH3 receptors, particularly those affecting the EGFR domain, have a potential association with age-related cSVD. Src and Notch have been demonstrated to exert a notable suppressive influence on the lumen and tube formation of endothelial cells (Sun et al., 2022). Furthermore, melatonin was shown to mitigate MMP-9-induced BBB damage through the NOTCH3/NF-κB pathway (Qin et al., 2019). Nevertheless, the precise manner in which these proteins express and regulate cSVD remains uncertain, and further research is required to elucidate this.

The GO enrichment analyses indicated the primary BPs linked to anti-cSVD genes were transcriptional regulation, regulation of kinase activity and protein phosphorylation; the main CC was receiver complex; the main MFs were protein kinase activity and kinase binding. For instance, transcription factors such as SRF and MYOCD are highly expressed in Alzheimer’s disease. These interactions are instrumental in orchestrating VSMC differentiation phenotypes and are of paramount importance for the regulation of blood flow in the cerebral circulation (Chow et al., 2007). It has been demonstrated that the inhibition of GLUT1 activates AMP-activated protein kinase, which exerts a pivotal impact on the angiogenesis of the central nervous system (Veys et al., 2020). The phosphorylation of protein kinase C-α and the subsequent activation of p115RhoGEF and RhoA is of significant importance in the dysfunction of the cerebral microvascular endothelial cell barrier induced by TNF-α (Peng et al., 2011). It can therefore be surmised that a number of biological functions, including transcriptional regulation, protein kinase activity and protein phosphorylation, are related to the role of Rhodiola in cSVD.

The KEGG enrichment analysis yielded results indicating the potential involvement of several crucial signal pathways in the pathogenesis of cSVD. The PI3K-Akt, Ras, Rap1, and MAPK signaling pathways were singled out as being of particular interest. For example, the anti-apoptotic effects of 3-n-butylphthalide on neural cells of rats affected by cSVD have been documented via the PI3K/Akt pathway (Qi et al., 2019). In maintaining the integrity of brain microvascular endothelial cells and the BBB, the Ras signaling pathway plays a critical part (Jiang et al., 2017). The EPAC-Rap signal plays an important role in the regulation of the blood-retina and BBB (Ramos and Antonetti, 2017). Ruscogenin has been demonstrated to alleviate BBB dysfunction induced by cerebral ischemia via the MAPK pathway (Cao et al., 2016). Nevertheless, the exact manner in which Rhodiola produces its therapeutical action on cSVD through these signal pathways remains to be elucidated, and further research is required to address this issue.

Molecular docking results revealed the existence of 28 pairs of key targets and compounds with favorable binding affinity in 36 results. Among the identified targets, MMP9 demonstrated the strongest binding affinity with three representative compounds (Umbelliferone, p-Coumaric acid, and Ethyl gallate) of Rhodiola. The relationship between MMP9 and certain compounds has been corroborated by scientific studies. For example, umbelliferone has been demonstrated in rheumatoid arthritis by diminishing the synthesis of proinflammatory factors, including MMP-9 and MMP-2, and by impeding the inflammatory response, invasion, and migration of fibroblast-like synovial cells (Cai et al., 2022). Furthermore, p-coumaric acid has been demonstrated to diminish M1 macrophage markers, cardiomyocyte apoptosis, and the inflammatory response to ischemia-reperfusion damage in the heart (Li N. et al., 2021). Additionally, Ethyl gallate has been demonstrated to downregulate the mRNA expression of MMP-2 and MMP-9 of mammary cancer cells, thereby inhibiting cell invasion and proliferation (Cui et al., 2015). While there is evidence that MMP-9 may be involved in the pathogenesis of cSVD, there is currently no experimental data to confirm how Rhodiola can affect cSVD through MMP-9.

Finally, the experimental results of endothelial cell leakage and migration indicate that umbelliferone, an effective component of Rhodiola, can enhance the permeability and migration capacity of endothelial cells that have been damaged by OGD. Additionally, the WB and IF results suggest that umbelliferone may protect tight junction proteins in endothelial cells. The experimentally validated targets and pathways include MMP9, MMP2, CCND1, PTGS2 and PI3K-Akt, Ras, and Rap1 signaling pathways. The results reveal the mechanism through which umbelliferone protects the endothelial barrier, thereby providing a foundation for the development of novel pharmaceuticals comprising umbelliferone and efficacious Rhodiola constituents, as well as data support for clinical applications.

While this study provides promising *in vitro* evidence, it is important to acknowledge the limitations of our findings. (1) Endothelial dysfunction is only one aspect of cSVD, which involves additional complex processes such as neuroinflammation, microcirculatory dysfunction, and white matter lesions. Future studies should explore how umbelliferone of Rhodiola may influence other pathophysiological mechanisms of cSVD beyond endothelial protection. (2) *In vivo* studies using cSVD animal models are required to validate the therapeutic potential of umbelliferone of Rhodiola in treating cSVD and to evaluate its safety and efficacy in a more complex physiological environment. (3) Clinical trials will be necessary to determine the applicability of Rhodiola as a therapeutic agent in human patients with cSVD.

## 5 Conclusion

In conclusion, it is the inaugural study to examine the mechanistic actions of umbelliferone, an efficacious component of Rhodiola, in treating cSVD via the lens of network pharmacology, molecular docking, and experimental validation. The above outcomes suggested the main active ingredients of Rhodiola were identified as Umbelliferone, Tyrosol, Ethyl gallate and p-Coumaric acid. The key targets are as follows: AKT1, EGFR, SRC, MMP9, MMP2, PTGS2, ESR1, CCND1 and GSK3B. And the main pathways are as follows: Notable among the pathways are the PI3K-Akt, Ras, Rap1, and MAPK signaling pathways. More importantly, cell experiments have shown that umbelliferone can safeguard the brain microvascular endothelial barrier through the mediation of MMP9, MMP2, CCND1, PTGS2 and multiple signaling pathways, including PI3K-Akt, Ras, and Rap1. In a word, our study provides a comprehensive and systematic clarification of the underlying mechanisms of umbelliferone of Rhodiola in treating cSVD, which exhibits multi-pathway and multi-target properties. The findings offer valuable insights and a foundation for further research in this field.

## Data Availability

The original contributions presented in the study are included in the article/Supplementary Material, further inquiries can be directed to the corresponding author.

## Abbreviations

ADME, absorption distribution metabolism and excretion; BBB, blood brain barrier; BC, betweenness centrality; BP, biological process; CC, closeness centrality; CC, cellular component; CSVD, cerebral small vessel disease; C-T, component target; ETCM, encyclopedia of traditional Chinese medicine; GI, gastrointestinal; GO, gene ontology; KEGG, kyoto encyclopedia of genes and genomes; MF, molecular function; PPI, protein-protein interaction; SMILES, simplified molecular input line entry system; TCM, traditional Chinese medicine; TCMSP, traditional Chinese medicine systems pharmacology.
